# Social activity, cognitive decline and dementia risk: a 20-year prospective cohort study

**DOI:** 10.1186/s12889-015-2426-6

**Published:** 2015-10-24

**Authors:** Riccardo E. Marioni, Cecile Proust-Lima, Helene Amieva, Carol Brayne, Fiona E. Matthews, Jean-Francois Dartigues, Helene Jacqmin-Gadda

**Affiliations:** Department of Public Health and Primary Care, University of Cambridge, Cambridge, CB2 0SR UK; Department of Psychology, Centre for Cognitive Ageing and Cognitive Epidemiology, University of Edinburgh, 7 George Square, Edinburgh, EH8 9JZ UK; INSERM, Centre INSERM U897, F-33000 Bordeaux, France; University Bordeaux, ISPED, Centre INSERM U897, F-33000 Bordeaux, France; Service de Neurologie, Department of Clinical Neurosciences, CHU, Pellegrin, 33076 Bordeaux, France; MRC Biostatistics Unit, Institute of Public Health, Cambridge, CB2 0SR UK

**Keywords:** Social engagement, Social network, Dementia, Cognitive decline, Longitudinal modelling

## Abstract

**Background:**

Identifying modifiable lifestyle correlates of cognitive decline and risk of dementia is complex, particularly as few population-based longitudinal studies jointly model these interlinked processes. Recent methodological developments allow us to examine statistically defined sub-populations with separate cognitive trajectories and dementia risks.

**Methods:**

Engagement in social, physical, or intellectual pursuits, social network size, self-perception of feeling well understood, and degree of satisfaction with social relationships were assessed in 2854 participants from the Paquid cohort (mean baseline age 77 years) and related to incident dementia and cognitive change over 20-years of follow-up. Multivariate repeated cognitive information was exploited by defining the global cognitive functioning as the latent common factor underlying the tests. In addition, three latent homogeneous sub-populations of cognitive change and dementia were identified and contrasted according to social environment variables.

**Results:**

In the whole population, we found associations between increased engagement in social, physical, or intellectual pursuits and increased cognitive ability (but not decline) and decreased risk of incident dementia, and between feeling understood and slower cognitive decline. There was evidence for three sub-populations of cognitive aging: fast, medium, and no cognitive decline. The social-environment measures at baseline did not help explain the heterogeneity of cognitive decline and incident dementia diagnosis between these sub-populations.

**Conclusions:**

We observed a complex series of relationships between social-environment variables and cognitive decline and dementia. In the whole population, factors such as increased engagement in social, physical, or intellectual pursuits were related to a decreased risk of dementia. However, in a sub-population analysis, the social-environment variables were not linked to the heterogeneous patterns of cognitive decline and dementia risk that defined the sub-groups.

**Electronic supplementary material:**

The online version of this article (doi:10.1186/s12889-015-2426-6) contains supplementary material, which is available to authorized users.

## Background

Increased social engagement and cognitive lifestyle activities have been associated with a decreased risk of cognitive decline and dementia [1–4]. Such markers are particularly promising from a public health perspective due to their modifiable nature [5]. However, due to the heterogeneity in measuring both social activity and cognitive ability, and with methodological challenges of analysing longitudinal population-based data, there is no consensus over which measures of social activity are the most important, which cognitive domains they affect (e.g., memory, processing, general ability), and if they have differential effects depending on the stage of decline (e.g., dementia [6] or mild cognitive impairment [7]). For example, a study by Seeman et al. [8] found frequent contact with family and friends and positive social support to be associated with better performance on tests of episodic memory and executive function. A 20-year follow-up study of Swedish data found mid-life political and mental activities, but not physical, social, cultural, or organizational activities to be associated with cognitive ability in later life [9]. Another study by Green et al. [10] found cross-sectional but not longitudinal associations between social networks and cognition.

One potential issue with models of social activity and cognitive decline is the possibility of reverse causation. People who have begun to decline and are entering a dementia phase at the start of a study may already have experienced a reduction in their social contacts [11, 12]. Further variation is introduced upon considering analytical issues common to longitudinal analyses of cognitive data. These include measurement error of cognitive function, dropout due to death and dementia, practice effects, and non-Gaussian distribution of cognitive test scores. Recently, we described two modelling approaches that can accommodate some of these issues – multi-state models and joint latent class mixed models [13].

Here we examine four markers of lifestyle activity and self-perception of social relationships (social, physical and intellectual engagement, social network size, satisfaction with social relationships, and perceived understanding) with longitudinal cognitive change and risk of dementia. A previous analysis on the same dataset combined these factors to create an overall index that was related to cognitive ability but not decline in the six psychometric tests examined [12]. The individual facets have also been modelled against risk of dementia [14]. However, the variables have not been studied in isolation with respect to cognitive decline, and using advanced modelling methods that include multivariate repeated cognitive information and consider risk of dementia alongside decline. First, instead of considering change separately in six individual cognitive tests [12], here we combine this multivariate information to model a single marker of general cognitive ability: the latent common factor underlying the tests. We relate the four social markers to change over time of this general cognitive ability and to the risk of dementia, which are modelled independently in the whole population. We then summarize the heterogeneity of cognitive ageing into several statistically defined homogeneous sub-populations derived from a joint latent class model that both accounts for shape of cognitive decline and dementia risk. We investigate whether lifestyle activity, self-perception of relationships are differently distributed within these homogeneous sub-populations.

## Methods

### Study population

Data are from the Paquid cohort, which is a longitudinal study of ageing with up to 20 years of follow-up [15]. Recruitment of 3777 participants occurred across 75 civil parishes of the Gironde and Dordogne regions of south west France. Subjects were aged 65 years and above, residing at home at the study baseline in 1988. There have been up to nine subsequent waves of data collected on participating individuals at 1, 3, 5, 8, 10, 13, 15, 17, and 20 years after the baseline assessment. At the first follow-up, only subjects from Gironde were interviewed.

### Consent

The ethical committee of the Bordeaux University Teaching Hospital approved the research according to the principles embodied in the declaration of Helsinki in 1988. However, all participants and their proxies were informed by the study investigators about the ongoing research activity and left free to accept/refuse their participation. Written informed consent was obtained from participants.

### Cognitive assessment and dementia diagnosis

Cognitive ability was assessed using tests of global cognitive function (the Mini-Mental State Examination – MMSE [16]), verbal fluency (the sum score of four trials from the Isaac’s Set Test truncated at 15 s [17]), abstract thinking (the sum score from the first five pairs from the Wechsler Similarities Test [18]), episodic memory and learning (Wechsler Paired Associate Test [19]), processing speed (Digit Symbol Substitution Test [18]), and immediate visual memory (the Benton Visual Retention Test [20]). The tests were administered at each wave by trained neuropsychologists.

Incident dementia was diagnosed at each wave by a trained psychologist based on the Diagnostic and Statistical Manual of Mental Disorders, third edition, revised (DSM-III-R). Individuals with suspected dementia were further examined by a neurologist to confirm the diagnosis.

### Covariates

The social activity and potential confounder variables were observed at the study baseline. Age was used as the continuous time-scale in the model.

Four markers of late-life engagement and self-perception of social relationships were assessed: social, intellectual and physical engagement (*engagement*); size of social network (*network size*); satisfaction with social relationships (*satisfaction*); and self-perception of feeling well understood (*understood*). Twelve questions were used to create a scale for late-life social, intellectual, and physical engagement. Seven of the questions had a binary response; the other five were measured on a four point Likert scale. The binary response questions included playing sport, travelling, visiting family and friends, looking after others e.g., grandchildren, and participation in a club, association or a ‘golden age’ club. The Likert response questions asked about the following activities: reading, watching television, knitting or doing odd jobs, playing board games, and gardening. A dichotomous response was created by combining the Likert responses “yes without difficulty” and “yes, but with difficulty” into a yes category and “no, because of difficulties” and “no, for other reasons” into a no category. A multiple correspondence analysis showed no evidence for the partition of the data into separate factors (Additional file 1: Figure S1) hence a general engagement variable was created by splitting the sum score of the twelve questions into tertile-based groups (0–4 activities, 5–7 activities, or 8–12 activities). Size of social network was dichotomised about the median into large (≥8 people) versus small (<8 people). A subject’s satisfaction with their relationships was assessed using a 4 response Likert scale, which was also dichotomised into satisfied and dissatisfied. A subject’s perception of how they feel others understand them was also split into two groups – well understood and not well understood. Categorical coding was used for adjustment covariates: Instrumental Activities of Daily Living (IADL), depression, sequelae of stroke, ischemic heart disease (IHD), diabetes (all coded “present” versus “absent”, IADL was coded as none versus any), sex, marital status (4 categories: married, divorced/separated, widowed, or single) and education (3 categories: no education up to a non-validated primary school degree; a validated primary degree up to a non-validated secondary degree; and a validated secondary degree or higher).

### Statistical analysis

Excluding individuals with no cognitive data or covariate data resulted in an analysis sample of 2854 participants (see flow chart in Additional file 1: Figure S2 for details).

#### Longitudinal cognitive change and dementia risk

Initially, cognitive decline and time to dementia were modelled independently. Evolution of the latent general cognitive factor underlying repeated measures of six cognitive tests was assessed using a linear mixed model with a quadratic age trend to account for non-linear mean cognitive decline [21]. Age (re-scaled by subtracting 65 years from all data points) was used as the time scale for the model. Given that a previous longitudinal analysis of cognitive decline in the Paquid cohort found no evidence for cohort effects [13], age at baseline was not included as a covariate. Random-effects were included to account for the individual variation in the intercept and both the linear and quadratic slopes of decline. Education and sex were considered as covariates. All the covariates included an effect on the baseline cognitive level and both the linear and quadratic slopes of decline. To remove a learning effect that has been observed in Paquid [22], cognitive decline was considered from first follow-up onwards [12]. A Cox proportional hazards model investigated the associations between the social activity variables and risk of dementia. Education, sex, and age at baseline were included as covariates. Schoenfeld residuals were examined to test the proportional hazards assumption. A sensitivity analysis also included further covariate adjustment for IADLs, depression, sequelae of stroke, IHD, diabetes, and marital status.

#### Joint latent class model

The joint latent class mixed model [23] (pictured in Additional file 1: Figure S3) combines the two sub-models: the linear mixed model on the underlying latent general cognitive factor and the proportional hazard model for time-to-dementia. Change in the general cognitive factor and dementia onset are linked through unobserved latent classes, which account for heterogeneity and represent sub-populations with different shapes of cognitive decline and risks of dementia.

Three sub-populations (latent classes) of cognitive ageing were identified by running this joint model with class-specific effects of sex and education on cognitive decline and class-common effects of sex and education in the survival (time-to-dementia) model. Full details of the modelling procedures are contained in Appendix 1 of the Additional file 1. Distribution of the lifestyle activity and self-perception variables across these classes were compared *a posteriori* using chi-squared tests for the categorical variables and analysis of variance (ANOVA) for the continuous measures.

Data were analysed using a Fortran90 programme (http://www.isped.u-bordeaux.fr/BIOSTAT) developed by Proust-Lima et al. [23] and in R [24] using the survival package [25].

## Results

The analysis cohort (*n* = 2854) is described in Table 1. The gender distribution was 41 % male and the majority of the cohort (57 %) had a mid-level education with 11 % having a validated secondary degree or higher qualification. The mean age of the sample at baseline was 77.0 (standard deviation (SD) 6.8) years and 783 were given a dementia diagnosis and 2200 died without dementia over the 20-year follow-up (mean time-to-diagnosed-dementia of 9.4 (SD 5.1) years). A low level of *engagement* was reported in 31 % of individuals, compared to 46 % and 23 % with medium and high levels, respectively. According to our classifications, around half of the cohort (48 %) had a large *social network*, under one fifth were *satisfied* with their social network, and 78 % felt that they were well *understood* by others.

**Table 1:** Description of characteristics for the whole Paquid cohort and the three classes of subjects defined by the joint latent class analysis

	Non-decliners		Moderate decliners		Fast decliners		All		
	(*N* = 1997)		(*N* = 611)		(*N* = 246)		(*N* = 2854)		
	N	%	N	%	N	%	N	%	P*
Demographics									
Sex (male)	857	43	235	38	90	37	1182	41	0.65
Education									0.06
Low	650	33	180	29	79	32	909	32	
Medium	1112	56	377	62	145	59	1634	57	
High	235	12	54	9	22	9	311	11	
Marital status									0.11
Married	1160	58	355	58	148	60	1663	58	
Divorced/Separated	692	35	202	33	78	32	972	34	
Widowed	87	4	31	5	18	7	136	5	
Single	58	3	23	4	2	1	83	3	
Social activity									
Social engagement									0.11
Low	593	30	208	34	89	36	890	31	
Medium	940	47	275	45	104	42	1319	46	
High social	464	23	128	21	53	22	645	23	
Large social network	966	48	298	49	113	46	1377	48	0.91
Satisfied with social relations	331	17	128	21	58	24	517	18	0.47
Feel understood by others	1592	80	456	75	179	73	2227	78	0.49
Dementia and death									
Dementia diagnosis^a^	157	8	423	69	203	83	783	27	<0.001
Deaths^a^	1488	75	485	79	227	92	2200	77	<0.001
Health variables									
Ischemic heart disease	421	21	127	21	39	16	587	21	0.59
Stroke	80	4	36	6	24	10	140	5	0.22
Diabetes	155	8	48	8	26	11	229	8	0.69
Depression	203	10	94	15	51	21	348	12	0.10
IADLs	484	24	164	27	86	35	734	26	0.21
	Mean	SD	Mean	SD	Mean	SD	Mean	SD	*P* ^†^
Demographics									
Age (years)	77.2	7.1	77.1	6.0	74.3	4.8	77.0	6.8	<0.001
Cognitive test data									
MMSE	26.8	2.7	25.1	4.5	23.4	6.8	26.2	3.9	<0.001
BVRT	11.0	2.4	10.0	2.7	9.9	3.1	10.7	2.6	<0.001
DSST	29.8	12.0	24.7	11.4	24.3	11.9	28.4	12.1	<0.001
IST	28.5	6.0	25.8	6.7	23.9	7.1	27.8	6.4	<0.001
WPA	4.2	1.6	3.7	1.6	3.7	1.7	4.0	1.6	<0.001
SIM	6.8	2.9	5.7	3.1	5.5	3.3	6.5	3.0	<0.001

*MMSE* Mini-mental state examination, *BVRT* Benton Visual Retention Task, *DST* Digit Symbol Substitution Test, *IST* Isaac’s Set Test, *WPA* Wechsler Paired Associates, *SIM* Similarities, *IADLs* Instrumental Activities of Daily Living, *SD* standard deviation. All variables were assessed at the study baseline with the exception of dementia diagnosis and death

^*^P-value for the chi-squared test for independence between education, sex, engagement/perception variables, and additional covariates in the three latent classes

^†^ANOVA P-value for differences in continuous traits in the three latent classes

^a^Dementia diagnosis and survival were assessed during the study follow-up

### Longitudinal cognitive change and dementia risk

In the whole population there was weak evidence for quadratic cognitive decline over time (Table 2). Having more education and being *engaged* in social, physical, and intellectual pursuits were associated with higher baseline cognitive ability. There was also an association between a higher educational level and slower linear cognitive decline but faster quadratic change (a compression of cognitive morbidity). Individuals who felt well *understood* had a slightly lower initial cognitive score but declined less over time than those who did not feel well *understood*.

**Table 2:** Mixed model output for the latent general cognitive factor from the one class model (*n* = 2854)

		Beta	SE	*P*
Intercept (I)		0		
Linear slope (t)		−0.47	0.46	0.30
Quadratic slope (t^2^)		−2.45	1.56	0.12
Female	I	0.04	0.19	0.83
	t	0.05	0.25	0.85
	t^2^	−0.42	0.81	0.60
Medium education	I	1.07	0.13	<0.001
	t	0.41	0.15	0.01
	t^2^	−1.58	0.53	0.003
High education	I	2.03	0.19	<0.001
	t	0.23	0.21	0.26
	t^2^	−0.75	0.71	0.29
Medium social engagement	I	0.57	0.30	0.06
	t	−0.13	0.48	0.79
	t^2^	0.11	1.66	0.95
High social engagement	I	0.70	0.29	0.02
	t	−0.07	0.45	0.87
	t^2^	−0.17	1.52	0.91
Large network size	I	0.11	0.12	0.37
	t	0.05	0.21	0.80
	t^2^	−0.38	0.71	0.59
Network satisfaction	I	−0.05	0.17	0.77
	t	0.03	0.23	0.90
	t^2^	−0.44	0.77	0.57
Feel well understood	I	−0.15	0.09	0.09
	t	0.14	0.06	0.03
	t^2^	0.00	0.06	0.94

The latent general cognitive ability is centered to 0 at time 0 in the reference category (intercept = 0 not estimated) and standardized to the between-subject variability at intercept

**Fig. 1:**
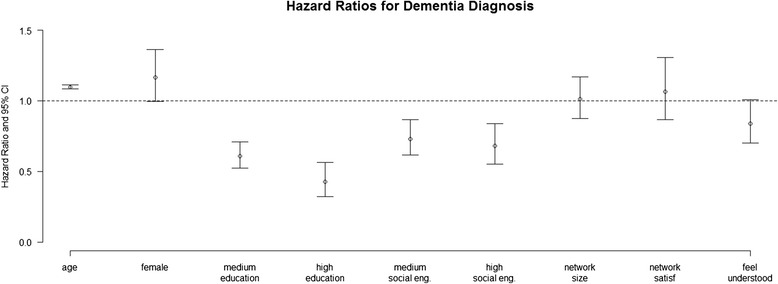
Hazard ratios for dementia for demographic and engagement/perception variables. All estimates are from multivariate models adjusting for age, sex, education, social/physical/intellectual engagement, network size, satisfaction with relationships within the social network, and a sense of feeling well understood by social contacts

In the time-to-dementia model (Fig. 1 and Additional file 1: Table S1) there was a decreased dementia risk for those with a medium or high level of *engagement* and a borderline-significant association for those who felt well *understood*. In the fully adjusted model (Additional file 1: Table S2), only the association for high level of *engagement* in social, physical, and intellectual pursuits was retained - Hazard Ratio (HR) 0.79 95 % confidence interval: 0.63, 0.99. Of the additional covariates, being widowed, having had a stroke, or having impairment with activities of daily living were all associated with an increased risk of dementia.

### Three homogeneous sub-populations of longitudinal cognitive change and dementia risk

**Fig. 2:**
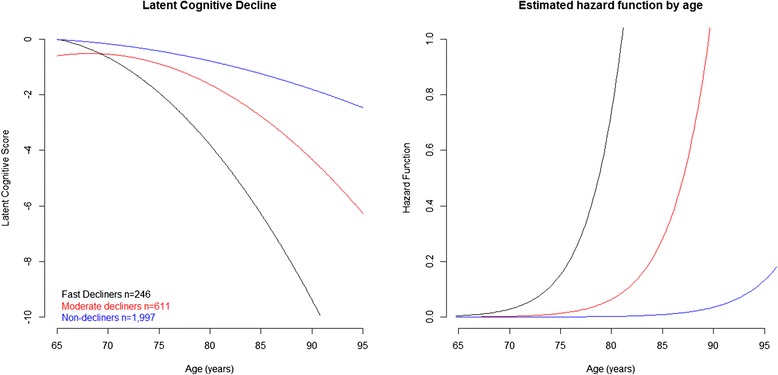
Latent general cognitive factor trajectories and risk of dementia in the three latent class model

The sex- and education-adjusted cognitive trajectories of the three latent classes are presented in Fig. 2 (left panel). The largest class contained 1997 individuals who did not show many signs of cognitive decline (*non-decliners*). The middle class (*n* = 611) showed moderate levels of cognitive decline (*moderate decliners*) while the smallest class (*n* = 246) had an accelerated rate of decline relative to the other two groups (*fast decliners*). The percentage of subjects diagnosed with dementia during the follow-up differed across the three groups (*n* = 157 (8 %), 423 (69 %), and 203 (83 %), respectively – *P* <0.001). The class-specific hazard functions displayed in Fig. 2 (right panel) highlight the large differences in the risk of dementia between the three sub-populations. Table 1 shows that there were few differences between the baseline characteristics of the groups, except for cognitive ability, which decreased from the *non-decliners* to the *moderate* decliners to the *fast decliners* (*P* < 0.001). The *fast decliners* were slightly younger than the other groups (mean age 74.3 (SD 4.8) years, *P* <0.001). None of the social-environment variables explained the heterogeneity in cognitive decline and dementia risk that was seen across the three sub-populations.

## Discussion

This study examined the associations between late-life measures of engagement in activities, self-assessed quality of relationships and longitudinal cognitive change and dementia onset over 20 years of follow-up. Two statistical modelling approaches were compared - a model where decline and dementia onset were modelled independently in the whole population, and an approach that categorised the population into three homogeneous underlying sub-groups.

Within the whole population, increased levels of *engagement* in social, physical, or intellectual pursuits were linked to increased baseline cognitive ability but not decline, and self-perception of feeling well *understood* was associated with a decreased linear cognitive decline. Increased levels of *engagement* and feeling well *understood* were also associated with a decreased risk of dementia in the whole sample but this relationship seems to be largely explained by marital and health status at baseline. In the sub-populations analysis, we identified a group of *fast decliners* with a high risk of dementia, a group of *moderate decliners* with a medium risk of decline and dementia, and a group of *non-decliners* with a low risk of dementia. None of the four social-environment variables helped to explain the heterogeneity in cognitive decline and dementia risk across the three sub-populations. There was also no evidence for baseline differences in the health and demographic variables across the three groups, with the exception of age. The group of *fast decliners* were, on average, slightly younger than the other two groups. The latent class model highlights sub-groups that decline faster or slower than subjects of the same age, sex and educational level. The *fast-decliners* group includes subjects with pathological decline compared to subjects of the same age.

There are several strengths to our analysis including the large sample size and long (20 year) follow-up of the population-based Paquid cohort, and the extensive cognitive test battery that was administered at each interview wave. Another point to highlight is the simultaneous use of the six psychometric tests, which precludes the requirement of a reference marker of decline. However, the key strength of this paper is the utility of the statistical approach, which included analyses based on both an independent and a dependent relationship between longitudinal cognitive change and dementia. The latter approach found evidence for three sub-populations within the global population that had different cognitive trajectories and risks of dementia.

A potential limitation of the analysis was the single time-point (baseline) measure of the social activity variables, which are dynamic in nature. Moreover, some of the variables that were included in the social, physical, and intellectual *engagement* measure might have been solitary activities - such as gardening and watching television – and so less compatible under the global banner of a social activity. Different levels of detail were also available for the various measures of social activity. For example, we had much finer detail available for the engagement marker (data from 12 questions) compared to the single question responses for network size, perceived understanding, and satisfaction with relationships. It is interesting to note that there were 157 individuals diagnosed with dementia in the *non-decliners* sub-group. This may have represented late-onset dementia relative to the start of the study. Indeed, this appears to be the case when we compare the mean time to dementia event in each sub-group (6.2 versus 10.5 versus 11.8 years in the *fast decliners*, *moderate decliners*, and *non-decliners*, respectively). Alternatively, some participants may be diagnosed with dementia despite exhibiting a relatively modest cognitive decline. A previous study in Paquid showed that this is more likely for those with low educational attainment [26].

Placing our results in context with previous findings is not straight-forward. As mentioned in the introduction, different studies invariably measure different indices of social activity and classify them using different approaches. It is also difficult to isolate activities that rely solely on social components with no physical or cognitive elements [4, 7–11]. Previous analyses undertaken in the same cohort as the present study (Paquid) found travelling, gardening, and doing odd jobs or knitting to associate with up to a 54 % decrease in risk of dementia over a 3 year period [27]. Furthermore, remaining or becoming socially/physically active over a 10 year period had up to a 49 % decrease in dementia risk over the subsequent 10 years [28]. This finding was also over and above any effect of cognitive decline over the initial 10-year period. Being satisfied with relationships and receiving more support than was given over the life-course have also been associated with a 23 % and 55 % decreased risk of dementia, respectively [14]. A recent Paquid analyses found an association between a general measure of social engagement with baseline cognitive ability but not decline in subjects without dementia [12], and a decreased risk of dementia in those who regularly participated in board games [29].

## Conclusions

We confirm associations between increased social engagement and higher cognitive ability scores but not cognitive decline. We also showed that in a large, population-based cohort, there is heterogeneity with respect to cognitive change and risk of dementia over a 20-year period. However, the four measures of social activity that we considered were not associated with this heterogeneity.

## Additional file

Additional file 1:
**Figure S1.** Multiple Correspondence Analysis of Social, Physical, and Intellectual Engagement Variables. **Figure S2.** Flowchart describing the baseline Paquid population. **Figure S3.** Schematic of the joint latent class mixed model of cognitive change and dementia. Supplementary Methods. (DOCX 268 kb)
