# Supplementary material for: Social activity, cognitive decline and dementia risk: a 20-year prospective cohort study
Source: BMC Public Health. 2015 Oct 24;15:1089. doi: 10.1186/s12889-015-2426-6 (PMC4619410; doi:10.1186/s12889-015-2426-6)
Supplement: Additional file 1: — Figure S1. Multiple Correspondence Analysis of Social, Physical, and Intellectual Engagement Variables. Figure S2. Flowchart describing the baseline Paquid population. Figure S3. Schematic of the joint latent class mixed model of cognitive change and dementia. Supplementary Methods. (DOCX 268 kb) [file 12889_2015_2426_MOESM1_ESM.docx]

Supplementary Figure 1: Multiple Correspondence Analysis of Social, Physical, and Intellectual Engagement Variables.

Supplementary Figure 2: Flowchart describing the baseline Paquid population.


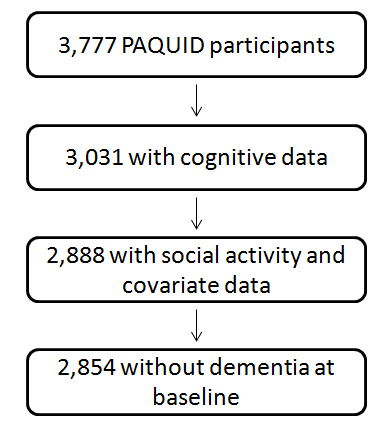


Supplementary Figure 3: Schematic of the joint latent class mixed model of cognitive change and dementia.

MMSE: Mini-mental state examination, BVRT: Benton Visual Retention Task, DSST: Digit Symbol Substitution Test, IST: Isaac's Set Test, WPA: Wechsler Paired Associates: SIM: Similarities
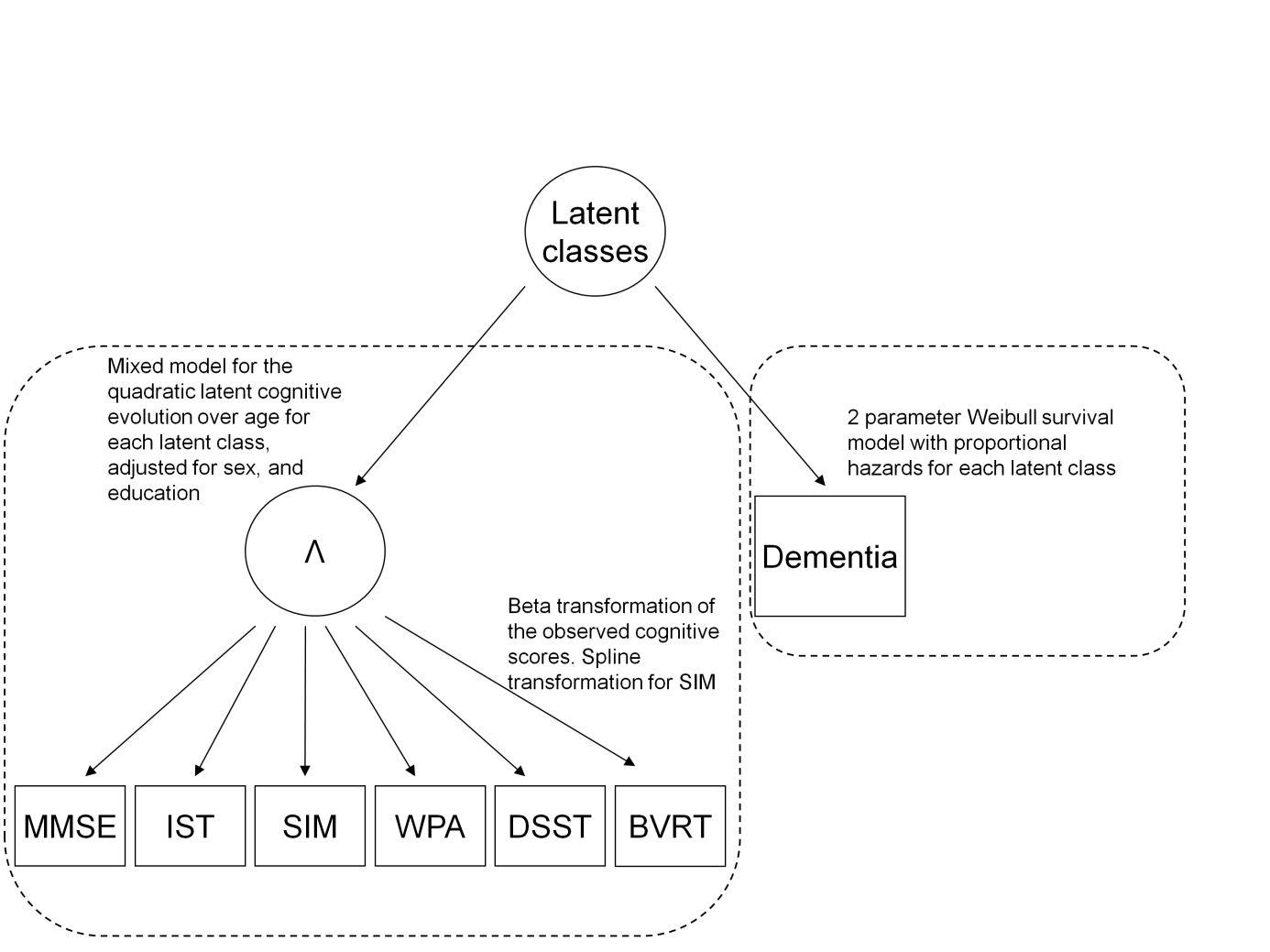
**Appendix 1: Supplementary Methods**

*Methodology of the two-step joint latent class model*

Given the computational complexity of the modelling, a two-step analysis was carried out.

First, the number of latent classes of cognitive decline and risk of dementia was determined. A joint model was built with dementia as the time-to-event outcome and evolution of latent cognitive function as the longitudinal process [1]. The time-to-event model was defined as a proportional hazard model with a two-parameter Weibull baseline risk function and class-specific proportional hazards. Sex and education were included as covariates and were assumed to be common across classes. The latent cognitive level was estimated using scores from the six cognitive tests. Each test was considered as a noisy measure of the true underlying cognitive ability. The model took into account the varying sensitivity of the tests to change (curvilinearity) that can lead to floor and ceiling effects and a non-Gaussian distribution of the score [2,3]. Curvilinearity was corrected by linking the observed scores with the true latent ability (named latent general cognitive factor) with parameterized nonlinear link function (a spline transformation with 4 (quartile-based) nodes for SIM and non-linear Beta Cumulative distribution functions for the five other tests). A random-effect term was included in each transformation to allow different individuals with the same latent cognitive ability to have different observed scores at the six cognitive tests. Evolution of latent cognitive ability within each latent class was assessed using linear mixed models with quadratic age trends to account for non-linear cognitive decline. Random-effects were included to incorporate individual variation in the intercept and both the linear and quadratic slopes of decline. Class-specific covariate effects for education and sex were included.

After parameter estimation, the subjects were assigned to the class to which they had the highest posterior probability to belong [1]. The number of latent classes was determined using the BIC selection criteria [1], entropy [4, 5], and by assessing the mean of the posterior probabilities of belonging to each latent class according to the final classification.

The model fit output is presented in Table A1. Although the BIC criteria suggests an improved fit for the 4 class model, the entropy value and posterior classification probabilities (mean posterior probabilities to belong to each latent class for subjects classified in this class) favour the 3-class model.

**References**

1. Proust-Lima C, Joly P, Dartigues JF, Jacqmin-Gadda H: Joint modelling of multivariate longitudinal outcomes and a time-to-event: A nonlinear latent class approach. Computational statistics & data analysis, 2009;53(4):1142-1154.

2. Proust-Lima C, Dartigues JF, Jacqmin-Gadda H: Misuse of the linear mixed model when evaluating risk factors of cognitive decline. Am. J. Epidemiol., 2011;174(9):1077-1088.

3. Morris MC, Evans DA, Hebert LE, Bienias JL: Methodological issues in the study of cognitive decline. Am. J. Epidemiol., 1999;149(9):789-793.

4. Muthén B, Brown CH, Masyn K, Jo B, Khoo ST, Yang CC, Wang CP, Kellam SG, Carlin JB, Liao J: General growth mixture modeling for randomized preventive interventions. Biostatistics, 2002;3(4):459-475.

5. Proust-Lima C, Séne M, Taylor JM, Jacqmin-Gadda H: Joint latent class models for longitudinal and time-to-event data: A review. Stat Methods Med Res 2014;23(1):74-90.

Table A1: Summary of Model Fit

| N classes | BIC | Mean posterior probabilities to belong to the latent class in which subjects are finally classified | Class Size | Entropy |
| --- | --- | --- | --- | --- |
| 1 | 254,762.7 | 1 | 2854 | 1 |
| 2 | 253,448.3 | 0.89, 0.93 | 494, 2360 | 0.83 |
| 3 | 252,921.3 | 0.86, 0.81, 0.83 | 246, 611, 1997 | 0.66 |
| 4 | 252,706.8 | 0.85, 0.79, 0.65, 0.74 | 181, 342, 1018, 1313 | 0.58 |

| N | 2,842 | | | |  |
| --- | --- | --- | --- | --- | --- |
| N events | 783 | | | |  |
|  |  |  |  |  |  |
|  | **HR** | **LCI** | **UCI** | **P** |  |
| Baseline age (years) | 1.10 | 1.08 | 1.11 | <0.001 |  |
| Female | 1.17 | 1.00 | 1.36 | 0.055 |  |
| Medium Education | 0.61 | 0.53 | 0.71 | <0.001 |  |
| High Education | 0.43 | 0.32 | 0.56 | <0.001 |  |
| Medium Social Engagement | 0.73 | 0.62 | 0.87 | <0.001 |  |
| High Social Engagement | 0.68 | 0.55 | 0.84 | <0.001 |  |
| Large Network Size | 1.01 | 0.88 | 1.17 | 0.879 |  |
| Network Satisfaction | 1.06 | 0.87 | 1.30 | 0.548 |  |
| Feel well understood | 0.84 | 0.70 | 1.01 | 0.062 |  |

Supplementary Table 1: Cox proportional hazards output for the association between engagement/perception variables and time to dementia diagnosis adjusted for age , sex, and education.

Supplementary Table 2: Cox proportional hazards output for the association between engagement/perception variables and time to dementia diagnosis adjusted for age , sex, education, and additional covariates.

| N | 2,842 | | | |  |
| --- | --- | --- | --- | --- | --- |
| N events | 783 | | | |  |
|  |  |  |  |  |  |
|  | **HR** | **LCI** | **UCI** | **P** |  |
| Baseline age (years) | 1.09 | 1.08 | 1.11 | <0.001 |  |
| Female | 1.13 | 0.95 | 1.34 | 0.17 |  |
| Medium Education | 0.64 | 0.55 | 0.74 | <0.001 |  |
| High Education | 0.45 | 0.33 | 0.60 | <0.001 |  |
| Medium Social Engagement | 0.85 | 0.71 | 1.01 | 0.07 |  |
| High Social Engagement | 0.79 | 0.63 | 0.99 | 0.04 |  |
| Large Network Size | 1.04 | 0.90 | 1.20 | 0.58 |  |
| Network Satisfaction | 1.05 | 0.85 | 1.29 | 0.65 |  |
| Feel well understood | 0.85 | 0.71 | 1.03 | 0.10 |  |
| Divorced/Separated | 1.03 | 0.87 | 1.22 | 0.74 |  |
| Widowed | 1.55 | 1.12 | 2.16 | 0.01 |  |
| Single | 1.09 | 0.72 | 1.66 | 0.68 |  |
| Ischemic Heart Disease | 0.89 | 0.74 | 1.07 | 0.21 |  |
| Stroke | 1.70 | 1.26 | 2.30 | <0.001 |  |
| Diabetes | 1.02 | 0.77 | 1.36 | 0.89 |  |
| Depression | 1.21 | 0.98 | 1.49 | 0.08 |  |
| Instrumental Activities of Daily Living (IADLs) | 1.39 | 1.15 | 1.65 | <0.001 |  |
